# Integrating Genome-Wide CNVs Into QTLs and High Confidence GWAScore Regions Identified Positional Candidates for Sheep Economic Traits

**DOI:** 10.3389/fgene.2020.00569

**Published:** 2020-06-17

**Authors:** Jie Cheng, Xiukai Cao, Quratulain Hanif, Li Pi, Linyong Hu, Yongzhen Huang, Xianyong Lan, Chuzhao Lei, Hong Chen

**Affiliations:** ^1^Key Laboratory of Animal Genetics, Breeding and Reproduction of Shaanxi Province, College of Animal Science and Technology, Northwest A&F University, Yangling, China; ^2^Computational Biology Lab, National Institute for Biotechnology and Genetic Engineering, Faisalabad, Pakistan; ^3^Pakistan Institute of Engineering and Applied Sciences, Islamabad, Pakistan; ^4^Key Laboratory of Adaptation and Evolution of Plateau Biota, Northwest Institute of Plateau Biology, Chinese Academy of Sciences, Xining, China

**Keywords:** copy number variations, QTL, GWAS, GWAScore, sheep economic traits

## Abstract

Copy number variations (CNVs) are important source of genetic variation, which can affect diverse economic traits through a variety of mechanisms. In addition, genome scan can identify many quantitative trait loci (QTLs) for the economic traits, while genome-wide association studies (GWAS) can localize genetic variants associated with the phenotypic variations. Here, we developed a method called GWAScore which collected GWAS summary data to identify potential candidates, and integrated CNVs into QTLs and high confidence GWAScore regions to detect crucial CNV markers for sheep growth traits. We got 197 candidate genes which were overlapping with the candidate CNVs. Some crucial genes (*MYLK3*, *TTC29*, *HERC6*, *ABCG2*, *RUNX1*, etc.) showed significantly elevated GWAScore peaks than other candidate genes. In this study, we developed the GWAScore method to excavate the potential value of candidate genes as markers for the sheep molecular breeding.

## Introduction

Chaka sheep also named Qinghai plateau half-fine wool sheep has been a geographical symbol of agricultural products by Ministry of Agriculture. Chaka sheep is famous for its excellent mutton quality, hence also known as the tributary sheep in the ancient times. Besides, its wool is exceptional for making woolen sweaters. Thus, Chaka sheep is reared as a multipurpose breed. Moreover, Hu sheep is one of the major breeds for lamb skin in China, whereas, the Small Tailed Han Sheep (STHS) depict high growth speed with good stress resistance.

Extensive studies have been conducted to associate phenotypes with two forms of genetic variations including single nucleotide polymorphisms (SNPs) and insertion/deletions (Indels). In recent years, copy number variation (CNVs) has emerged as a potential source of genetic divergence, bearing significant phenotypic and economically important traits, however, CNVs have not been thoroughly characterized so far. CNV is defined as a DNA segment of ∼50 bp or larger in size and present submicroscopic copy number variation in comparison with a reference genome, including deletions, insertions, duplications, and complex multi-site variants (Redon et al., 2006; Mills et al., 2011). Presently, in order to detect the CNVs in a genome, various genome-wide platforms have been developed, including SNP genotyping platforms (Di Gerlando et al., 2018), array comparative genomic hybridization (aCGH; Zhang et al., 2014), and next-generation sequencing (NGS) (Xu et al., 2017). For sheep, SNP arrays and CGH arrays are routinely used, and their performances in CNV detection have been reviewed in sheep in various studies (Fontanesi et al., 2011; Liu et al., 2013). These methods have moderated power in detecting duplication regions, which have been proven to be affected by cross-hybridization of repetitive sequence and low probe density (Bickhart et al., 2012). NGS is well accepted in the genome-wide identification of CNV.

Quantitative trait loci (QTLs) is genetic basis of economic trait, such as muscle weight in carcass, body weight, milk protein percentage. However, such traits are mostly based on multiple genomic variants. Since the genes are pleiotrophic, multi-traits GWAScore can improve the confidence of crucial gene variants (Flint et al., 2014). Candidate gene method is one of the traditional methods to identify the important gene. It is easy to use, and the effect of candidate gene on livestock phenotypes can be inferred based on the known-function genes in the model animal (Cheng et al., 2019). However, for genes with unknown functionality, this method could not possess reliable results. At present, 3D genomics is a powerful method to identify crucial CNV impacting phenotypes (Lupiáńez et al., 2015; Will et al., 2017; Zhang et al., 2018). However, the costs of NGS and the downstream analysis are significantly high. Our method identifies CNVs using resequencing data which then integrates with the existing genome-wide association studies (GWAS) and QTL data. QTL are regions often associated with one or more important traits conferring phenotypes. GWAS can identify significant genetic variants associated with one or more interest phenotypes. GWAScore could quantitate the probability distribution of key variants in genome regions.

Single nucleotide polymorphisms have been extensively used for GWAS data, but integrating GWAS data based on CNVs for sheep has been rarely reported. Here, we used NGS to detect the CNVs in sheep. As a result, we have developed a new method called GWAScore to look for important regions across the genome by summarizing the GWAS data, integrating QTLs and high confidence GWAScore regions with CNVs to identify positional candidates for sheep breeding to accelerate genetic improvement.

## Materials and Methods

### Ethics Statement

The protocols were approved by the Faculty of Animal Policy and Welfare Committee of Northwest A&F University (FAPWC-NWAFU, protocol number, NWAFAC1008).

### Sample Collection and Sequencing

Chaka, Hu, and STHS are three distinct breeds from China, which were collected from Qinghai, Jiangsu, and Shandong provinces, respectively (detail in Supplementary Table S1). DNA were extracted from whole blood samples of Chaka sheep (*n* = 10) as previously described (Pan et al., 2018). The libraries were subjected to NGS with the HiSeq 4000 platform with paired-end mode. Trimmomatic (Leading:20 Trailing:20 Slidingwindow:3-15 Avgqual:20 Minlen:35 Tophred:33) and FASTQC software were employed to trim the sequences to assess the quality of the raw sequence data. Sequence reads were mapped, sorted and deduped to the reference sheep genome^1^ by BWA-MEM (0.7.13-r1126) and Picard v2.18.2^2^. The command of BWA-MEM “mem -t 12 -M -R” was used for each individual read. The PICARD SortSam and MarkDuplicates were also used to sort and dedupe. The average sequencing depth was 7.68× for the 10 Chaka sheep. Raw sequencing data of Hu and STHS have been submitted to the NCBI Sequence Read Archive with accession number SRP066883 (Pan et al., 2018). Sequencing data of Ten Chaka sheep have been submitted to NCBI (project number PRJNA594811).

### Whole-Genome Copy Number Variation Identification

To assess the copy number variations of sheep including Chaka (*n* = 10), Hu (*n* = 10) and Small Tailed Han (*n* = 9) Sheep, we first genotyped the 29 individuals that represent three important Chinese sheep breeds. The CNVs were identified using CNVcaller methods utilizing NGS data (Wang et al., 2017). The CNVs were calculated in 1000 bp sliding window with 500 bp steps along the autosomes. Nextly, the absolute number of copies in each window were calculated and reads with high similarity (≥97%) were merged to determine the copy number variation regions. Primary CNVR were further processed into merged CNVR for genotyping of the data set. We combined the identified CNVs among all individuals.

### Integrating CNV with GWAS and QTL Data

To detect significant loci with phenotypic effects, we integrated GWAS and QTL aggregate with CNV. GWAS and QTL data about growth traits, were retrieved from Animal QTLdb^3^. All data were mapped to sheep genome Oar_v4.0 and visualized using CIRCOS.

Array density, phenotypic measurement errors, and statistical methods lead to poor reproducibility of GWAS (Barendse, 2011; Schaid et al., 2018; Wijmenga and Zhernakova, 2018). In our study, GWAScore was developed to study genomic regions which impact the phenotypic effects using GWAS data. The method excels these factors by concisely summarizing GWAS SNPs of different studies and QTLs. Here, GWAScore was calculated with 100 kb window (Cao et al., 2019) as

$${\text{GWAScore}}_{\left(x,x+0.1\right)}=\frac{-n_{g\,w\,a\,s\,\left(x,x+0.1\right)}\times \sum_{i=1}^{n_{s\,n\,p\,\left(x,x+0.1\right)}}\log P}{0.1\times n_{s\,n\,p\,\left(x,x+0.1\right)}}$$

n*_*gwas (*x*, *x* + 0.1)*_* is the number of GWAS in the genomic region (*x*, *x* + 0.1), n*_*snp (*x*, *x* + 0.1)*_* is the number of SNPs in genomic region (*x*, *x* + 0.1), *P* is the *P* value of significant SNPs. Whereas, (*x*, *x* + 0.1) means the positions of each windows, and the size of window was 0.1 Mb. GWAScore derived from the significance SNP loci was used to quantify the probability of key mutations in genome (GWAScore > 0).

### Gene Ontology and Kyoto Encyclopedia of Genes and Genomes Pathway Analysis

It was in QTLs and high confidence GWAScore regions that identified genome-wide CNVs and then annotated positional candidate gene for sheep growth traits. Entering that, Gene Ontology (GO) and Kyoto Encyclopedia of Genes and Genomes (KEGG) pathways were completed (*P* < 0.01) by metascape online software^4^ (Zhou et al., 2019).

## Results

### The Analysis of CNVs

Copy number variations (CNVs) are an important source of genetic variations. We obtained 4301 CNV regions in total by using the whole genome sequencing data from the three breeds. The average length of the CNVs was about 4 kb. Among them, we identified 19%, 18% and 56% CNVs located in exonic, intronic and intergenic regions, respectively (detail in Supplementary Table S2). Furthermore, 4301 CNVs were commonly shared by all the breeds in the current study.

**FIGURE 1:**
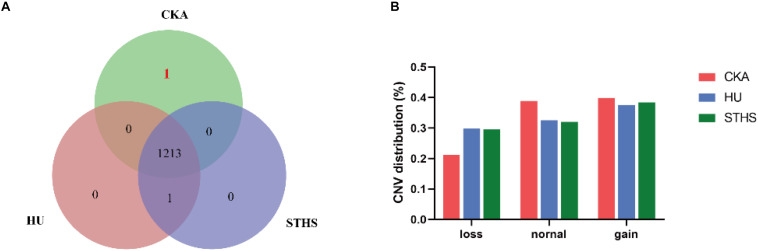
The analysis of CNVs. **(A)** breed-specific CNV genotype in different breeds. **(B)** CNV distribution in these three breeds.

CNV caller was used to genotype CNVs into “dd, Ad, AA, AB, BB, BC, and M.” Moreover, “dd and Ad” were grouped into “Loss genotype,” “AA” into “Normal genotype” and “AB, BB, BC, and M” into “Gain genotype.” If all individual of one breed have the same CNV genotypes which didn’t appear in other two breeds, we will take it as a breed-specific CNV. Then, we found the number of breed-specific CNV genotype was very low and only one specific CNV genotype for Chaka sheep. The specific and shared CNV genotypes are represented in a venn diagram for all the three breeds (Figure 1A). The only one breed-specific CNV in Chaka sheep was found at chr11: 27042001-27044500. CNVs are distributed into loss, normal and gain mutations among the three breeds and are presented in Figure 1B.

### Integrative Analysis Annotates Candidate Genes of Sheep Economic Traits

A total of 4301 CNV regions, 729 carcass QTLs (detail in Supplementary Table S3) and 440 GWAS markers were collected. GWAScore was designed to use independent GWAS to calculate the phenotypic effects of a given genomic region. The GWAScore map of 190 regions ranging from 1.30 to 16.55, covering 19.0 Mb of the genome was presented (Supplementary Table S4).

**FIGURE 2:**
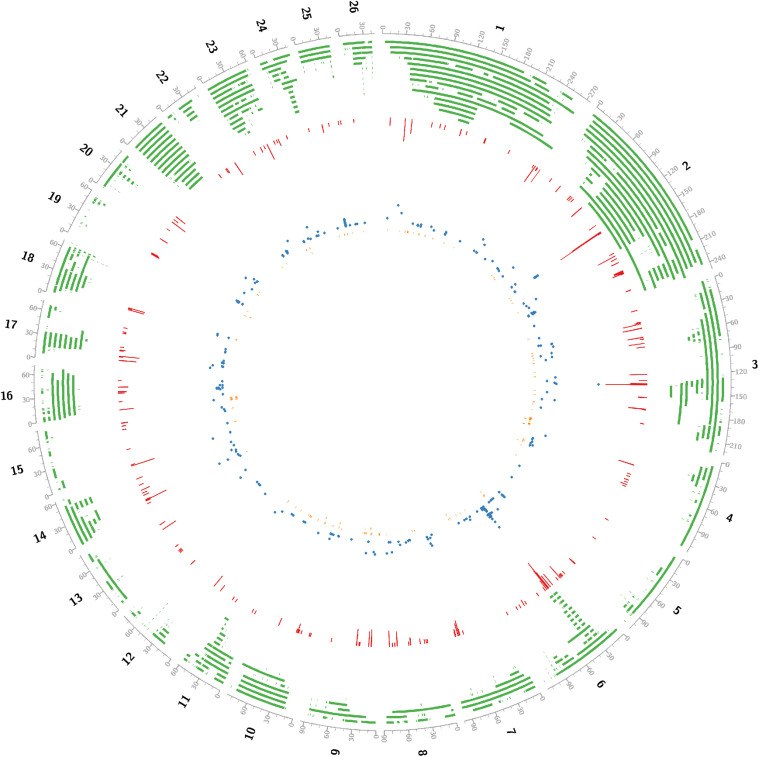
Concentric circles showed that integrating gnome-wide CNVs into QTLs and high confidence GWAScore regions identified positional candidates for sheep growth traits. black line, genome; green line, tile_QTL in sheep; red line, histogram_GWAScore; blue line, scatter_GWAS; orange line, tile_ copy number variation in sheep. All positions were transformed into Oar_v4.0. Using Perl software, data from Animal QTLdb and NCBI.

Critical CNV markers were revealed by integrative analysis of 729 carcass QTL and 440 GWAS markers as shown in Figure 2, in which we identified 197 coding candidate genes located in common regions shared by GWAScore regions, QTL and CNVs regions. Some crucial candidate genes were identified with higher GWAScore such as myosin light chain kinase 3 (*MYLK3*), tetratricopeptide repeat domain 29 (*TTC29*), HECT and RLD domain containing E3 ubiquitin protein ligase family member 6 (*HERC6*), ATP binding cassette subfamily G member 2 (*ABCG2*) and Runt-related transcription factor 01 (*RUNX1*), which may be associated with important economic traits. Among the 197 candidate genes, we identified 23 CNVs located in the exon of gene (*HERC6*, etc.) and five CNVs located in the upstream or 5’UTR of gene (*ABCG2*, etc.) (Table 1, detail in Supplementary Table S5).

**TABLE 1:** Integrative analysis annotated candidate genes (top five) of sheep traits.

Chromosome	Start	End	GWAScore	The number of QTL	Overlap genes	Location
chr14	14618001	14622000	7.815613972	4	*MYLK3*	intronic
chr17	11330501	11334500	6.915066425	1	*TTC29*	intronic
chr6	36222001	36224500	6.882224908	17	*HERC6*	exonic
chr6	36353001	36356000	6.882224908	17	*ABCG2*	UTR5
chr1	264845001	264848500	6.649751982	2	*RUNX1*	intronic

### GO and KEGG Analysis

**FIGURE 3:**
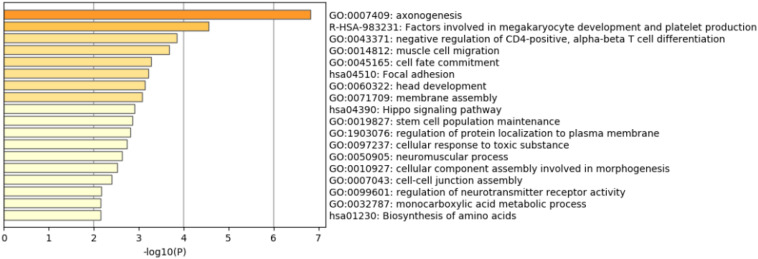
The most enriched GO and KEGG terms.

We analyzed 190 regions with GWAScores, and identified important CNVs to annotate 197 common genes. We dissected these 197 genes by GO and the KEGG Pathway Analysis (Figure 3). The genes enriched axonogenesis (GO:0007409), factors involved in megakaryocyte development and platelet production (R-HSA-983231), negative regulation of CD4-positive, alpha-beta T cell differentiation (GO:0043371), and muscle cell migration (GO:0014812).

## Discussion

Researchers have extensively utilized SNPs as genetic markers for GWAS, but the identification of CNVs has gathered the highlight for its associated with complex traits. Genome-wide association studies based on CNV markers have been reported in livestock, such as milk production traits, milk somatic cell score in Holsteins (Xu et al., 2014; Ben Sassi et al., 2016; Duran Aguilar et al., 2017), feed conversion ratio in beef cattle (de Almeida Santana et al., 2016), growth traits in *Bos indicus* (Zhou et al., 2016), quantitative traits in Brown Swiss cattle (Prinsen et al., 2017), and umbilical hernia in swine (Long et al., 2016). Although the well-powered GWAS provided by the integrated array of CNVs may be a perfect solution in livestock breeding, it didn’t integrate all the GWAS data from different papers and besides it is still not financially viable to serve the majority of the scientific community under a sea of samples (McCarroll and Altshuler, 2007). Integrating GWAS data based on CNVs for sheep are rarely reported. Cao et al. (2018) have integrated cattle genome CNV with various meta-QTL and identified CNV of *GBP4* as a positional candidate for cattle stature. But GWAS based QTL were omitted in their study which may provide additional information for phenotypic CNV identification (Cao et al., 2018).

Candidate gene method is one of the traditional methods to identify the important gene. The effect of candidate gene on livestock phenotypes can be inferred based on the known-function genes in the model animal (Cheng et al., 2019). For unknown-function genes, we can’t use candidate gene method. In this study, we used a novel method, developed by our group called GWAScore which could quantify the probability to be a QTL of a given region by GWAS data to reveal novel candidates. This method has helped to detect key genes associated with muscle development from transcriptome (Cao et al., 2019). QTLs, regions in the genome, contain genetic variants affecting the quantitative traits of interest. Genome-wide association studies is identifying genetic variants associated with one or more interest phenotypes (Georges et al., 2019). In our study, by using public data of GWAS from Animal QTLdb^3^, we integrated whole-genome CNVs into QTLs and high confidence GWAScore regions for whole-genome analysis.

These 197 candidate genes were analyzed by the GO Analysis (*P* < 0.01) and the KEGG Pathway. These genes were enriched in axonogenesis (GO:0007409), factors involved in megakaryocyte development and platelet production (R-HSA-983231), negative regulation of CD4-positive, alpha-beta T cell differentiation (GO:0043371), and muscle cell migration (GO:0014812), which showed that the function of these gene were associated with neural development, immunity regulation (Cloutier et al., 2018) and muscle migration, imposing strong signals toward the economic traits.

We extracted crucial genes with highest GWAScore and overlapped CNVs in these regions (Table 1, detail in Supplementary Table S5), including the well-known gene *MYLK3*, *TTC29*, *HERC6*, *ABCG2*, *RUNX1*, etc. *MYLK3* is the member of the myosin light chain (MYL) family. Importantly, myosin is the main component of the myofibrillary filament, which plays a crucial role in the growth and contraction of muscle (Zhang et al., 2009). *TTC29* is associated with muscle mass in informative regions of the QTL (Kärst et al., 2011), and *TTC29* has also been recognized as candidate genes for residual feed intake in pigs (Do et al., 2014). *HERC6* has significant effects on fat and protein yield (Cohen-Zinder et al., 2005). There is significant association between *ABCG2* polymorphisms and milk production trait in Holsteins, Chinese Holsteins, Brown Swiss cows, and Sheep (Cohen-Zinder et al., 2005; Yue et al., 2011; Árnyasi et al., 2013; Cecchinato et al., 2014). It was reported that *RUNX1* plays a vital role in neural development, hematopoiesis, and leukemogenesis (Speck and Gilliland, 2002; Yoshikawa et al., 2007). The GWAS hit SNP within *RUNX1* is associated with the mean corpuscular volume level in pigs. We identified 23 CNVs located in the exon of genes, such as *DLGAP1*, *ADGRB1*, and *AGPAT3*. DLG associated protein 1 (DLGAP1) which may regulate DLG1, is associated with control of infection by ovine lentivirus (White et al., 2012). Adhesion G protein-coupled receptor B1 (*ADGRB1*) gene has antagonistic or sensitive effects on *Escherichia coli* F17 resistance to diarrhea (Jin et al., 2018). 1-Acylglycerol-3-phosphate O-acyltransferase 3 (*AGPAT3*) gene may make triacylglycerol and phospholipid synthesis in milk biosynthesis (Bionaz and Loor, 2008).

## Conclusion

We used a new method called GWAScore and integrated whole-genome CNVs into QTLs and high confidence GWAScore regions, which identified potential candidate for the first time in sheep including the well-known gene *MYLK3*, *TTC29*, *HERC6*, *ABCG2*, *RUNX1*, etc.

## Data Availability Statement

Raw sequencing data of Hu and Small Tailed Han Sheep have been submitted to the NCBI Sequence Read Archive under accession number SRP066883. Sequencing data of Ten Chaka sheep have been submitted to NCBI under submission number SUB6671624 (project number PRJNA594811).

## Ethics Statement

The animal study was reviewed and approved by the Faculty of Animal Policy and Welfare Committee of Northwest A&F University (FAPWC-NWAFU). Written informed consent was obtained from the owners for the participation of their animals in this study.

## Author Contributions

JC and XC designed the project. JC wrote the manuscript. QH and XC revised the manuscript. LP and LH provided the sample of Chaka sheep. YH, XL, CL, QH, and HC provided the correction of the manuscript.

## Conflict of Interest

The authors declare that the research was conducted in the absence of any commercial or financial relationships that could be construed as a potential conflict of interest.

## Footnotes

1 https://www.ncbi.nlm.nih.gov/assembly/GCF_000298735.2/, Oar_v4.0

2 http://broadinstitute.github.io/picard/

3 http://www.animalgenome.org/

4 http://metascape.org/gp/index.html#/main/step1

## Abbreviations

*ABCG2*: ATP binding cassette subfamily G member 2
aCGH: array comparative genomic hybridization
*ADGRB1*: adhesion G protein-coupled receptor B1
*AGPAT3*: 1-acylglycerol-3-phosphate O-acyltransferase 3
CNVs: copy number variations
CNVR: copy number variation region
*DLGAP1*: DLG associated protein 1
GWAS: genome-wide association studies
*HERC6*: HECT and RLD domain containing E3 ubiquitin protein ligase family member 6
Indels: insertions/deletions
*MYLK3*: myosin light chain kinase 3
*MYL*: myosin light chain
NGS: next-generation sequencing
QTL: quantitative trait loci
*RUNX1*: Runt-related transcription factor 1
SNPs: single nucleotide polymorphisms
*TTC29*: tetratricopeptide repeat domain 29.
